# Investigating the feasibility of scale up and automation of human induced pluripotent stem cells cultured in aggregates in feeder free conditions^☆^

**DOI:** 10.1016/j.jbiotec.2013.12.009

**Published:** 2014-03-10

**Authors:** Filipa A.C. Soares, Amit Chandra, Robert J. Thomas, Roger A. Pedersen, Ludovic Vallier, David J. Williams

**Affiliations:** a Wellcome Trust – Medical Research Council Cambridge Stem Cell Institute, Anne McLaren Laboratory for Regenerative Medicine and Department of Surgery, University of Cambridge, UK; b Centre for Biological Engineering, Loughborough University, UK; c Wellcome Trust Sanger Institute, Hinxton, UK

**Keywords:** Induced pluripotent stem cells, Feeder free, Automation, Scale up, Manufacturing

## Abstract

The transfer of a laboratory process into a manufacturing facility is one of the most critical steps required for the large scale production of cell-based therapy products. This study describes the first published protocol for scalable automated expansion of human induced pluripotent stem cell lines growing in aggregates in feeder-free and chemically defined medium. Cells were successfully transferred between different sites representative of research and manufacturing settings; and passaged manually and using the CompacT SelecT automation platform. Modified protocols were developed for the automated system and the management of cells aggregates (clumps) was identified as the critical step. Cellular morphology, pluripotency gene expression and differentiation into the three germ layers have been used compare the outcomes of manual and automated processes.

## Highlights

• First published protocol for scalable automation of hiPSC in feeder-free conditions.
• Successful transfer of hiPSC between sites representative of research and manufacture.
• Comparability between manual and automated expansion protocols for hiPSC.

## 1 Introduction

Human induced pluripotent stem cells (hiPSC) are generated from reprogrammed fibroblasts by overexpression of pluripotency factors (Takahashi et al., 2007; Yu et al., 2007). These pluripotent cells have the unique characteristic to self renew in vitro while maintaining the capacity to differentiate into a broad number of cell types. By combining these unique properties, hiPSC could enable the generation of large quantity of cells for clinical applications. Furthermore, the possibility of generating hiPSC from somatic cells using epigenetic reprogramming represents a unique opportunity for personalized regenerative medicine. Indeed, these pluripotent stem cells could enable the production of patient specific cell types that are fully immuno-compatible with the original donor thereby avoiding the need for immune suppressive treatment after cell transplantation. Nevertheless, the practical, financial and temporal obstacles in producing and validating personalized clinical-grade hiPSC and their differentiated progeny will almost certainly limit the feasibility of this approach. These limitations could selectively restrict patient access to autologous cell-based therapies (Faden et al., 2003). The creation of clinical banks of hiPSC from donors that can provide HLA matching to recipients is proposed as a strategy to attenuate the host immune response to transplanted tissue (Lin et al., 2009; Taylor et al., 2005, 2011).

Similarly, hiPSC can be used to develop in vitro disease models, allowing large scale studies otherwise restricted due to the limited availability of primary cells and biopsy material. This application has been proven useful to model neurodegenerative diseases, cardiac syndromes and metabolic disorders in vitro for basic studies and drug screening (Ebert et al., 2009; Moretti et al., 2010; Rashid et al., 2010). However, each of these applications requires large quantity of hiPSC produced in reproducible and standardized ways. Indeed, expansion of hiPSC remains time and resources consuming while experimental variability due to human intervention is almost systematic.

Process automation has been a key mechanism to achieve controlled and standardized cell production. Successful automated protocols have been developed for the expansion of human mesenchymal stem cells (Thomas et al., 2007) and human embryonic stem cells (hESC) (Thomas et al., 2009b). Scale up automation enables scale out for conventional formats with predictable process variation and quality outcome by removing manual interventions. However, little work has been done in developing technologies for automation and scale up of hiPSC for healthcare applications. Several solutions and technologies have been developed for live cell production in suspension platforms (Ratcliffe et al., 2012). However these are not readily adapted for cells growing in adherent conditions. Furthermore, large scale production of hiPSC for clinical applications would require expansion in culture using clinically compatible conditions in a reproducible way without loss of function and in sufficient numbers to create reproducible and cost effective therapeutic products. Finally, passaging represent the main difficulty to develop an automation platform to expand hiPSC since these cells must be propagated as aggregates/clumps to maintain their integrity and quality (Beers et al., 2012). Indeed, evidence indicates that hiPSC grown and harvested as single cells are more likely to acquire genetic anomalies (Amps et al., 2011). Consequently, standardization of cell counting and cell clump size measurement has proven to be impractical. Here, we have addressed all these issues by transferring an established manual method to grow hiPSC in feeder free and chemically defined medium onto an automated platform compatible with large scale production. This study shows for the first time that large scale automated production of hiPSC is possible without the need of single cell dissociation thereby respecting their natural properties.

## 2 Materials and methods

### 2.1 Manual maintenance and passage of hiPSC in feeder free and chemically defined medium

**Fig. 1:**
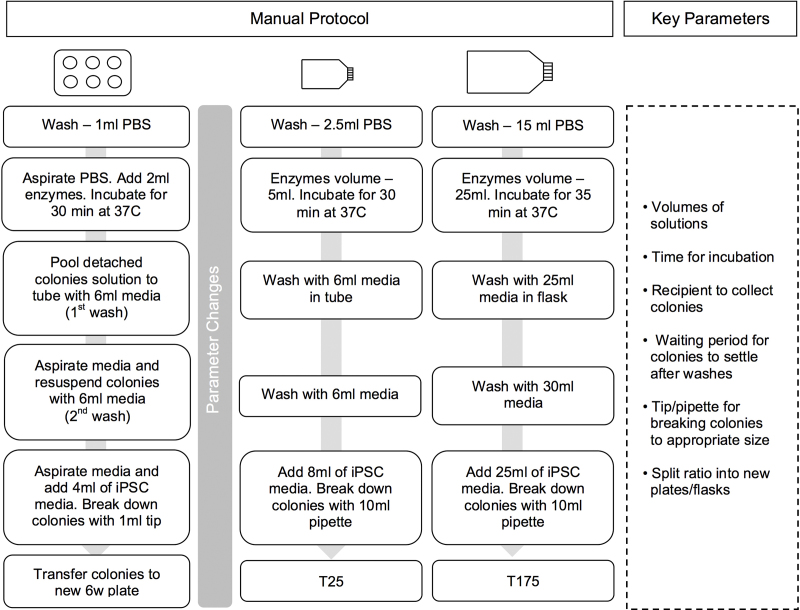
Protocol steps for manual passage of hiPSC using six well plates, T25 flasks and T175 flasks. The original six well plate protocol was scaled up for T25 and T175 flasks. Volumes of reagents and media were scaled up accordingly, the incubation time for T175 flasks increased and the 1 ml tip replaced by 10 ml pipettes. The key parameters were identified and are listed above.

hiPSC were cultured in feeder-free conditions using chemically defined medium (CDM-PVA) with Activin A (10 ng ml^−1^, R&D System) and FGF2 (12 ng ml^−1^, R&D Systems) – iPSC medium, as previously described (Brons et al., 2007). The composition of CDM-PVA was 50% IMDM (Gibco) added to 50% F12 + GlutaMax-1 (Gibco), supplemented with 1% lipid concentrate (Gibco), 7 μg ml^−1^ of insulin (Roche), 15 μg ml^−1^ of transferrin (Roche), 450 μM of monothioglycerol (Sigma) and 1 mg ml^−1^ of Polyvinyl Alcohol (Sigma). Cells were harvested after 6 or 7 days of culture (dependent on visually confirmed confluence) using 1 mg ml^−1^ collagenase IV (Gibco) and 1 mg ml^−1^ dispase (Gibco). Detached colonies were aspirated and pooled into a conical tube and washed with CDM-PVA medium. A second wash step was performed before colonies were gently broken down into smaller cell aggregates (clumps) by pipetting and allowed to settle under gravity. It must be noted here that the aim was only to reduce the size of the aggregates and not to reduce them to single cells. Cell clumps were plated at 1:10 split ratio to 0.1% porcine gelatin plates (Sigma), pre-coated with mouse embryonic fibroblast medium containing 10% FBS (Biosera) for 24 h at 37 °C and 5% (v/v) CO_2_. iPSC medium and 10 μM of Y27632 (Sigma) was added for the first 48 h. Following this, maintenance medium was replaced daily until readiness for the next passage. This protocol is routinely used for 6 well plates and T-25 flasks (Fig. 1).

### 2.2 Manufacturing platform and instrumentation

The CompacT SelecT (The Automation Partnership, UK) is a fully automated cell culture platform which incorporates a small six-axis anthropomorphic robotic arm (Cell Therapy Manufacturing Facility, 2011) that can access 90 T175 flask and plate incubators, controlled at 37 °C under an atmosphere of 5% (v/v) CO_2_ and humidity. The system allows the automation of seeding, feeding and other cell culture processes in order to maintain cell lines in standard T175 cell culture flasks. Flasks are bar-coded for identification and cell process tracking. Two flask decappers and flask holders, automated medium pumping and an automatic cell counter (Cedex^®^, Roche Innovatis AG, Germany) are integrated within a high-efficiency particulate air (HEPA) filtered cabinet to ensure sterility. At Loughborough University, the CompacT SelecT has been successfully used to culture many different cell types including human mesenchymal cells and human embryonic stem cells (hESC) (Thomas et al., 2007, 2009a,b). The CompacT SelecT has also been shown to be successful at preventing contamination when the GMP version of the CompacT SelecT passed the “sterile fill” runs (Chandra et al., 2012). The CompacT SelecT allows activities during cell culture such as seeding, media changes and measurement cells in a controlled environment (Thomas, 2012). Thus this platform can be used to expand and differentiate batches of cells to a tighter specification than manual cell culture (Liu et al., 2010).

### 2.3 Automated passage of hiPSC in feeder free and chemically defined medium using CompacT SelecT

The automation enables scale out for conventional formats with predictable process variation and quality outcome by removing manual interventions. The CompacT SelecT is a preferred platform for development process friendly method of automating the culture of cells that grow in adherent conditions. The automation step mimics the manual process and is therefore demonstrably similar to the manual cell culture steps. For many manual cell culture protocols, there is a centrifugation step to concentrate the cell suspension and allow for cells to be washed. However, in this instance, cells grow in aggregates and do not require centrifugation as they settle under gravity.

In order to transfer the culture protocol to the CompacT SelecT it was necessary to scale up from a T25 to a T175 flask, media volumes were scaled proportionally to flask surface area, and work within the restricted set of plasticware and the allowable positioning of the plasticware within the automated system (Fig. 1). Operating conditions of the manual culture process were followed as closely as possible (temperature, timing, splitting ratio, mixing, volumes), however a number of detailed changes had to be made to the manual protocol as are discussed later in the manuscript.

### 2.4 Differentiation of hiPSC into endoderm, mesoderm and neuroectoderm

hiPSC were plated into gelatin plates pre coated with 10% FBS and maintained for 24 h in iPSC medium before inducing differentiation into the three germ layers: endoderm, mesoderm and neuroectoderm as described previously (Vallier et al., 2009). Differentiation was induced by supplementing CDM-PVA with Activin (R&D System), bFGF (R&D System), BMP4 (R&D System), LY294002 (Promega), SB431542 (Tocris) and CHIR99021 (Stemgent) at different times and concentrations (Supplementary Online Material).

Supplementary material related to this article can be found, in the online version, at http://dx.doi.org/10.1016/j.jbiotec.2013.12.009.

Table S1 Detailed protocol for inducing differentiation of hiPSC in the three germ layers: endoderm, mesoderm and neuroectoderm. RPMI-B27 composition was 98% RPMI 1640 + GlutaMax (Gibco), 2% of B-27 supplement (Gibco) and 1% MEM non-essential amino acids (Gibco).

### 2.5 Immunochemistry

hiPSC were fixed for 20 min at 4 °C in 4% paraformaldehyde (PFA) and washed three times in PBS. Cells were incubated 1 h at room temperature in PBS containing 10% donkey or goat serum (depending on antibody, Serotec), for intra-cellular epitopes 0.1% Triton X-100 (Sigma) was added to the blocking solution. Cells were then subsequently incubated with primary antibodies diluted in 1% serum in PBS overnight at 4 °C. Dilutions were as follows: TRA-1-60 (Santa Cruz, 1:100), OCT4 (Santa Cruz, 1:100), NANOG (R&D Systems, 1:100), and anti-SOX2 (R&D Systems, 1:100), SOX17 (R&D Systems, 1:200), EOMES (Abcam, 1:100), BRAC (R&D Systems, 1:100), MIXL1 (Abcam, 1:100), Nestin (Abcam, 1:100). Cells were washed 3 times in PBS and incubated with secondary antibodies, Alexa Fluor 568 (Invitrogen) and Alexa Fluor 488 (Invitrogen), for 2 h at room temperature. Cells were washed three times with PBS and stained with Hoechst 33258 (Sigma, 1:10,000 diluted in PBS) for 5 min. The cells were viewed in PBS using a Zeiss Axiovert 200M microscope.

### 2.6 RNA extraction, reverse-transcriptase PCR and quantitative PCR

Total RNAs were extracted from hiPSC using the GenElute Mammalian Total RNA Miniprep Kit (Sigma). For each sample 0.5 μg of total RNA was reverse transcribed using Superscript II reverse transcriptase (Invitrogen). RNA and primer were denatured at 65 °C for 5 min and RT-PCR reaction mixtures incubated at 25 °C for 10 min, 42 °C for 50 min and 70 °C for 10 min. qPCR was performed using Sensi Mix Sybr Low Rox Kit (Bioline). A negative control that contained only water and a positive control that contained RNA extracted from human embryonic stem cells (H9-WiCell) were also ran. The expression of the PBGD housekeeping gene was used to normalize qPCR reactions.

## 3 Results and discussion

**Fig. 2:**
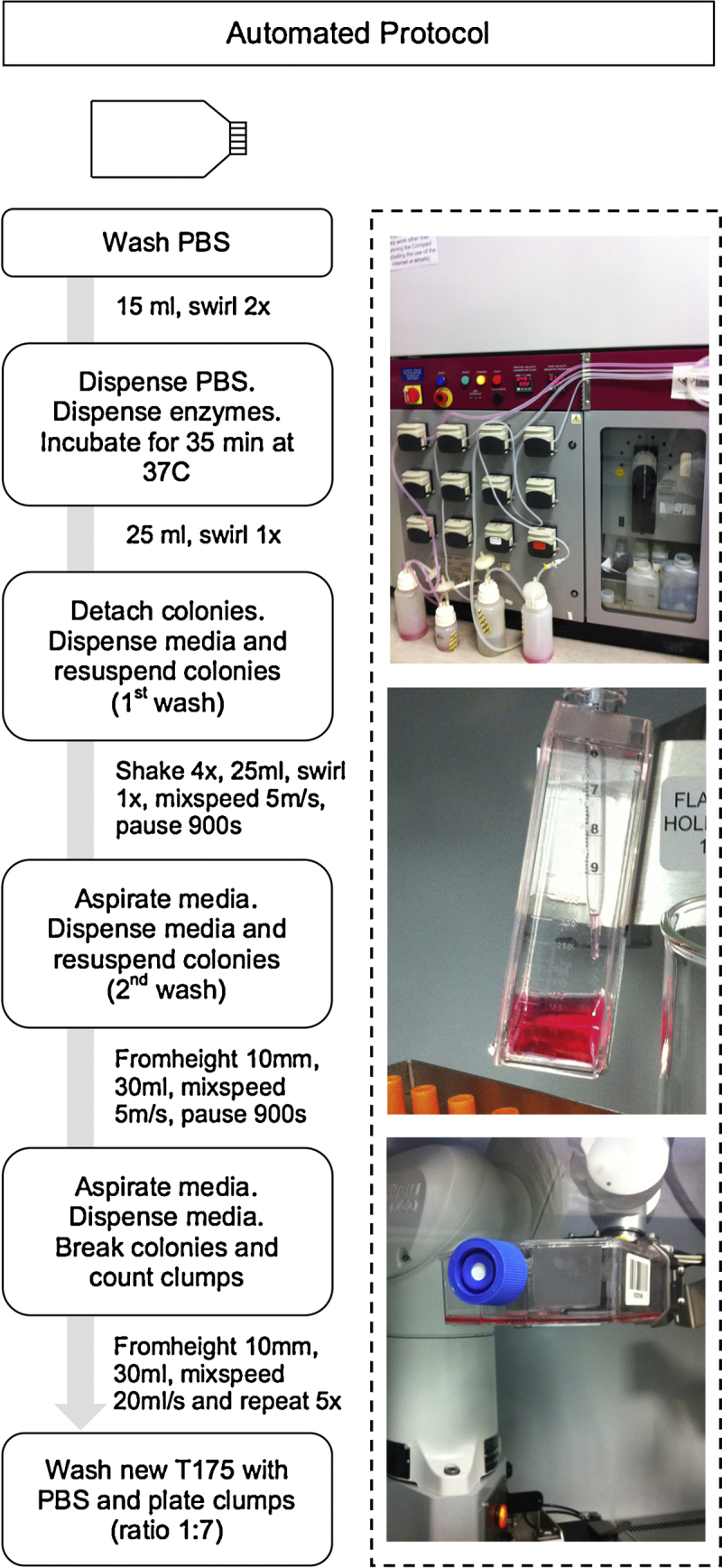
Protocol steps for automated passage of hiPSC using ComppacT SelecT. Selected protocol for automated passage of hiPSC from mature colonies to broken clumps passaged into new flasks.

An human induced pluripotent stem cell line – BBHX8 (Vallier et al., 2009), derived from adult human fibroblasts using retroviral reprogramming with OCT4, SOX2, KLF4 and c-MYC was derived and manually maintained at the Anne McLaren Laboratory for Regenerative Medicine (LRM), University of Cambridge. In order to develop an automation process to expand hiPSC using CompacT SelecT, the hiPSC line was transferred to the Centre for Biological Engineering (CBE), Loughborough University where it was further cultured and passaged in T25 flasks using the manual protocol established at LRM (Brons et al., 2007). This line was subsequent scaled up to T175 flasks (Fig. 1). When transferring to T175 flasks, two major limitations of the CompacT SelecT were taken into consideration: (i) as an alternative to the use of conical tubes the T175 flask was used to wash and pellet the cell clumps by gravity. This removed a process step but required a protocol modification that increased the time required for settling of aggregates. (ii) 10 ml pipettes were used instead of 1000 μl tips when breaking the cell clumps to a desirable size (Fig. 2). Cells were maintained using the manual protocol in both T25 and T175 flasks to show process transfer and scale up within both facilities.

The manual process of expanding T175 flasks was executed for three weeks to gain confidence in the protocol before cells were transferred to the CompacT SelecT. A split ratio of 1:10 was maintained and the cell clumps broken to the appropriate size by the operator using a 10 ml pipette (Table 1). However it become evident that mimicking the breaking of cell clumps to the desirable size by the operator would be the most difficult step to automate.

To adapt the T175 manual protocol to the CompacT SelecT, four iterations of the automation protocol were written and tested in the CompacT SelecT. The changes made in the four versions of the automated protocol were based on: (a) the time for the colonies to settle down by gravity after washing, (b) the distance of the pipette from the bottom of the flask when removing diluted enzyme solution without aspirating the cells clumps, (c) the pipetting speed and number of mixing steps required to achieve an homogeneous cell suspension of the desirable cell clump size without the presence of an undue number of single cells, and (d) the split ratio (Table 2). To take into account the small volumes of some reagents and to prevent inaccuracies of the dispensed volumes by a 10 ml pipette or 1.6 mm bore tubing, the media formulation was mixed manually. Therefore, the automation platform was used to dispense the complete media.

In the first version (*A*) of the automated protocol, several cell clumps were lost during the washing steps as the time for cell clumps to settle was insufficient, additionally several clumps were aspirated due to the pipette reaching close to the bottom of the flask when aspirating the enzymatic solution. Care is essential during the two washes in order to dilute the enzyme concentration without aspirating cell clumps. Moreover the low speed of mixing was insufficient to break the clumps to the desired size. A higher split ratio was used when comparing to manual protocol. This was chosen to account for any potential cell loss during the automation steps described in the above paragraph (a)–(c). The following versions (B–D) looked at improving *Version A* (Table 2) and subsequently the outcome of the passage.

It proved difficult to use the CEDEX cell counter to count clumps of cells in order to passage cells based on cell count as the CEDEX could not count the clumps accurately (data not shown). This would have improved process reproducibility, however the methods available for counting cell aggregates/clump are not yet suitable to be integrated within an automation platform.

A feature of the CompacT SelecT is its 90 T175 flask carousel incubators. While the robot placed the flasks gently in the incubator carousel without disturbing the cells, as soon as the carousel was rotated, cell clumps moved under centrifugal force to concentrate in parts of the flask resulting in a heterogeneous distribution of the cell clumps/colonies in the flask; this in particular can result in the growth of differentiated colonies. This observation showed the importance of leaving the unattached cells in an undisturbed condition and that even the slightest movement can result in a heterogeneous distribution of the colonies.

**Fig. 3:**
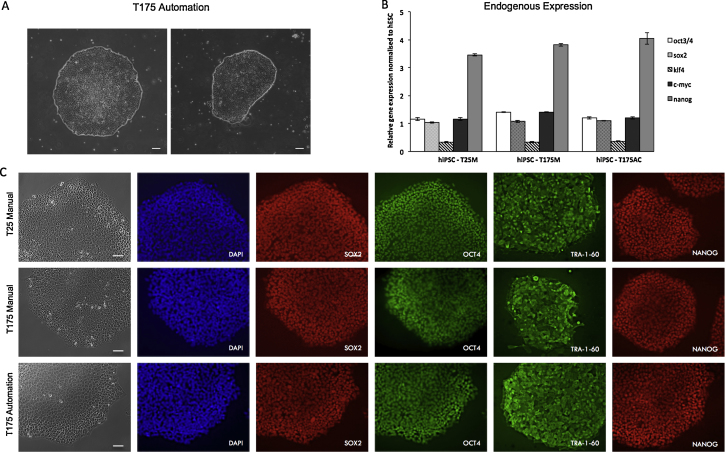
Characterization of hiPSC passaged using manual protocol in T25 flasks and T175 flasks and using automated passage in T175 flasks. (A) Bright field microscope images of hiPSC colonies passaged manually in T175 flasks using the CompacT SelecT. Scale bar = 60 μM. (B) Expression of pluripotency genes by qPCR for the hiPSC passaged manually in T25 flasks and T175 flasks and passaged using automation in T175 flasks. Three technical replicates were performed for each sample and all genes were normalized to PBGD and hESC. Scale bar = 20 μM. (C) Expression of pluripotency markers (SOX2, OCT4, TRA-160 and NANOG) analyzed by immunochemistry. Both analyses show no significant difference between the two manual and the automated protocol. Scale bar = 20 μM. *Abbreviation*: hESC, human embryonic stem cells.

Of the four flasks/protocol versions (A–D) tested in the CompacT SelecT, C was the best protocol based on colony morphology and absence of differentiation 7 days after passage (Fig. 2). To further assess the quality of these cells when compared to manual protocols (T25Manual and T175Manual), expression of pluripotency markers was analyzed using qPCR and immunochemistry. These analyses showed high levels and homogenous expression of pluripotency markers and morphology characteristic of hiPSC for both manual and automated protocols (Fig. 3).

**Fig. 4:**
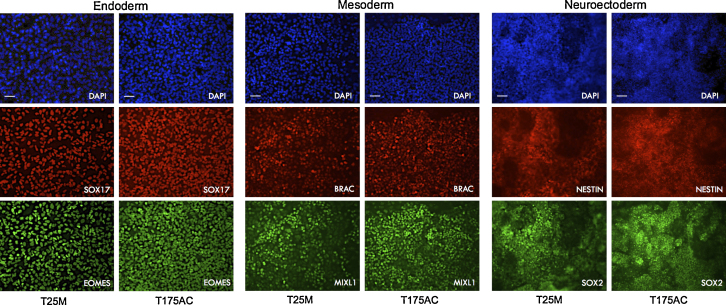
Immunostaining analysis of the expression of endoderm, mesoderm and neuroectoderm markers in hiPSC passaged using manual protocol in T25 flasks and using automated passage in T175 flasks. Expression of endoderm markers (SOX17 and EOMES), mesoderm markers (BRAC and MIXL1) and neuroectoderm markers (NESTIN and SOX2) in hiPSC for both manual and automated protocols. Scale bar = 20 μM. Abbreviation: T25M, T25 manual; T175AC, T175 automated.

In addition, capacity of differentiation toward the three germ layers was confirmed by growing the hiPSC into culture conditions inductive for endoderm, mesoderm and neuroectoderm differentiation. Both manual protocols in T25 flasks and the automated protocol in T175 flasks showed homogeneous differentiation of hiPSC into the three germ layers as observed by the change of cell morphology and by the expression of endoderm markers: SOX17 and EOMES, mesoderm markers: BRACHURY and MIXL1 and neuroectoderm markers: NESTIN and SOX2 (Fig. 4). These results suggest that cells expanded with our automation protocol retain the capacity to produce cell types with a clinical interest.

Considered together, these data suggest that the transfer of protocols between the two facilities was successful and that both scale-up and automation protocols were shown to maintain cell function comparable to manually passaged hiPSC.

## 4 Conclusion

In conclusion, we have successfully demonstrated a protocol passaging hiPSC in an automated system, the CompacT SelecT, with cells maintained as aggregates. This work has shown that hiPSC can be passaged in an automated system without losing their pluripotent capabilities. Cells maintained their characteristic hiPSC morphology and expressed pluripotency markers both by immunochemistry and qPCR. Additionally, these cells maintained the capacity to differentiate into the three germ layers (endoderm, mesoderm and neuroectoderm). The comparison between manual and automated conditions showed the maintenance and passaging of hiPSC is feasible using the CompacT SelecT system, however to improve reproducibility some detailed changes will have to be implemented. Further work is necessary to define more accurately the critical protocol parameters and take account of the constraints required when generating fully functional hiPSC lines for clinical applications.

With respect to future automated solutions incubator design must take into account the current necessity of not disturbing cells that require to be grown as aggregates, such as hiPSC and that take time (potentially up to 24 h) to attach. Automation would most certainly benefit from development of (conical) plastic vessels of the volume necessary to accommodate cell suspensions from T175 flasks as this could reduce the time for cell clumps to settle and thus speeding the passaging protocol. The option to use a pipette with smaller bore than a 10 ml pipette would assist the optimization of breaking cell clumps to the desirable size and reduce the single cell debris caused by pipetting. While these modifications would be required to optimize the present protocol to ensure maximum yields of cells, this study represents a proof of principle that hiPSC can be expanded in clumps using automation without loss of quality.

## Figures and Tables

**Table 1:** Changes made between protocols: T25 manual, T175 manual and T175 automated.

	Incubation time (min) | incubator temperature (°C)	Plasticware used to wash clumps	Time for colonies to settle down by gravity after washing (min)	Plasticware used to break clumps	Number of pipetting movements to break clumps	Split ratio
T25 manual	30 | 37.5	Conical tube	5	1000 μl tips	5	1:10
T175 manual	35 | 37.5	T175 flask	10	10 ml pipette	5	1:10
T175 automated	35 | 36.5	T175 flask	10–15	10 ml pipette	7	1:7–1:5

**Table 2:** The four versions of the automated protocol showing the parameters changed in the protocols.

	Time for colonies to settle down by gravity after washing (min)	Height from bottom of flask to aspirate enzymatic solution (mm)	Speed of mix and final dispense of liquid in pipette to break colonies in final step (ml/s)	Split ratio
Version A	10	0.5	5 | 1	1:7
Version B	15	5	10 | 1	1:7
Version C	15	10	20 | 1	1:7
Version D	15	10	50 | 50	1:5
