# Supplementary material for: Investigating the feasibility of scale up and automation of human induced pluripotent stem cells cultured in aggregates in feeder free conditions
Source: J Biotechnol. 2014 Mar 10;173(100):53–8. doi: 10.1016/j.jbiotec.2013.12.009 (PMC3969287; doi:10.1016/j.jbiotec.2013.12.009)
Supplement: Table S1 — Detailed protocol for inducing differentiation of hiPSC in the three germ layers: endoderm, mesoderm and neuroectoderm. RPMI-B27 composition was 98% RPMI 1640 + GlutaMax (Gibco), 2% of B-27 supplement (Gibco) and 1% MEM non-essential amino acids (Gibco). [file mmc1.pdf]

Lineage	Duration	Basal media	Cytokine	Concentration
**Endoderm**	Day 1	CDM-PVA	Activin-A	100 ng/mL
			FGF-2	80 ng/mL
			BMP4	10 ng/mL
			LY29004	10 μM
			CHIR99021	3 μM
	Day 2	CDM-PVA	Activin-A	100 ng/mL
			FGF-2	80 ng/mL
			BMP4	10 ng/mL
			LY29004	10 μM
	Day 3	RPMI-B27	Activin-A	100 ng/mL
			FGF-2	80 ng/mL
**Mesoderm**	3 days	CDM-PVA	Activin-A	100 ng/mL
			FGF-2	20 ng/mL
			BMP4	10 ng/mL
			LY29004	10 μM
			CHIR99021	5 μM
**Neuroectoderm**	11-12 days	CDM-PVA	FGF-2	12 ng/mL
			SB431542	10 μM
			Noggin	150 ng/mL
			SB431542	10 μM
